# Efficacy of Microneedle as an Assisted Therapy for Melasma: A Meta-analysis and Systematic Review of Randomized Controlled Trials

**DOI:** 10.1007/s00266-024-04395-2

**Published:** 2024-10-16

**Authors:** He Simin, Xue Siliang, Chen Wei, Diao Ping, Li Erlong, Zhao Jianbo

**Affiliations:** 1 Department of Dermatology, West China Hospital, Sichuan University, 37 Guoxue Lane, Wuhou District, Chengdu, 610041 Sichuan Province China; 2 Laboratory of Dermatology, Clinical Institute of Inflammation and Immunology, Frontiers Science Center for Disease-Related Molecular Network, West China Hospital, Sichuan University, Chengdu, 610041 China; 3 College of Information and Communication Engineering, Harbin Engineering University, Harbin, China

**Keywords:** Melasma, Microneedle, Microneedle-assisted therapy, Meta-analysis

## Abstract

**Background:**

Melasma is a common hyperpigmentary disorder, it has variety of treatment options, but it usually has a poor curative effect and high recurrence rate. Microneedles have shown certain prospects in the treatment of melasma as an assisted therapy, but there is no consensus on its efficacy and safety. To evaluate the efficacy and safety of microneedles as an adjuvant treatment for melasma.

**Methods:**

Statistical tools were used to adjust the improvement of MASI scores in all studies to obtain standardized mean differences (SMD), and then, meta-analysis were performed. Risk ratio (RR) was utilized to assess adverse reactions, clinical effectiveness, and patient satisfaction.

**Results:**

The effects of microneedle-assisted treatment for melasma begin to manifest at the 4th week, with optimal results observed at the 24th week, and with a high patient satisfaction. Compared with oral medication alone, microneedle-assisted therapy began to be more effective at week 12 and continued by 24 weeks. Compared with laser therapy alone, microneedle-assisted therapy also showed stronger efficacy, at the 8th week, microneedle-assisted treatment was significantly more effective, reaching its peak at the 12th week. Finally, in the comparison of microneedle and microinjection therapy, microneedle has always been more significant than micro-injection.

**Conclusions:**

Microneedle is a valuable adjunctive therapy for melasma treatment. It enhances long-term clinical outcomes compared to monotherapy and is associated with high patient satisfaction.

**Level of Evidence IV:**

This journal requires that authors assign a level of evidence to each article. For a full description of these Evidence-Based Medicine ratings, please refer to the Table of Contents or the online Instructions to Authors www.springer.com/00266.

**Supplementary Information:**

The online version contains supplementary material available at 10.1007/s00266-024-04395-2.

## Introduction

Melasma is an acquired pigmented spot, which is typically characterized by yellow-brown pigmented spots with clear boundaries in the middle of the face, cheekbones, and lower jaw. The common affected skin type is Fitzpatrick skin type III–IV. Melasma occurs mainly in child-bearing period female, with estimates ranging from 8.8 to 40% [1], and the rate of Asian women can be as high as 30% [2], which affects the patient’s appearance and causes serious psychological impact. The treatment of melasma includes prevention of sun exposure, repair of skin barrier, local and systemic medication, chemical peeling, and physical therapy [2]. But the treatment usually has a poor curative effect and high recurrence rate.

Microneedle is a procedure involving a small, needle-like instrument that creates micro-perforations in the skin, allowing for the direct delivery of drugs and active substances into the dermis, capillaries, and subcutaneous tissue [3]. Simultaneously, the minor damage induced by microneedle can stimulate the skin’s repair and regeneration processes, enhance collagen and elastin fiber proliferation, restore the skin barrier, and accelerate epidermal turnover [4]. Current studies have shown that microneedles have shown certain prospects in the treatment of melasma as an assisted therapy, but there is no consensus on its efficacy and safety.

This study aims to summarize the research on microneedles as an adjuvant treatment for melasma, use evidence-based medicine to verify their efficacy and safety, and provide a basis and strategy for the treatment of melasma.

## Materials and Method

This study was conducted based on the preferred reporting items for systematic reviews and meta-analyses (PRISMA) guidelines [5].

### Search Strategy

We performed a literature search to identify relevant articles available from web of science, PubMed, Cochrane central register of controlled trials, Embase, Sinomed, Wanfang data, Chinese National Knowledge Infrastructure and VIP databases. Using keywords chloasma, melasma, microneedling and microneedle without any language or date restrictions, from inception to January 2024. All randomized controlled studies on microneedle as an adjuvant therapy in the treatment of melasma were included, and references included in the studies were searched to prevent missed selection. Articles/titles/abstracts with these keywords were screened independently by two reviewers using pre-established inclusion criteria to identify relevant studies for inclusion in this meta-analysis.

### Eligibility Criteria

The included studies met the following criteria: (1) patients with facial melasma; (2) no other underlying diseases and no restrictions on race and sex; (3) all randomized controlled studies on microneedle as an adjuvant therapy in the treatment of melasma; (4) It must include melasma area and severity score (MASI) or its variations, and can also include clinical efficacy, patient satisfaction, adverse reactions.

The exclusion criteria were as follows: (1) studies that included the type of microneedle as radio frequency or soluble microneedle; (2) duplicate literature, meetings, case reports, reviews, and other mismatched literature; (3) no control group study; (4) evaluative criteria do not include MASI or its variations.

### Data Extraction and Quality Assessment

The data were independently extracted and cross-checked by two researchers. Any differences were settled by discussion and consensus. When the study data were incomplete, attempts was made to contact authors for missing or original data. If it was unavailable, the study was excluded.

The following information was extracted from each study: first author, publication year, country, study type, sample size, average age and age range, Fitzpatrick skin type, intervention methods, microneedle characteristics, dropout rate, adverse events, evaluate time, evaluation item and efficacy. The severity of melasma was assessed by MASI, which is based on subjective assessments of three factors: area of involvement, darkness and homogeneity, scoring ranges from 0 to 48 points [6]. Other variations of MASI, modified MASI, and hemi MASI, range from 0 to 24 points [7].

The quality of each study was independently assessed by two researchers using the risk of bias 2.0 (RoB 2.0) tool [8]. Any discrepancies were resolved through discussion or consultation with experts to mitigate potential biases arising from the subjective preferences of a single evaluator.

### Statistical Analysis

The indicators used to assess the improvement of melasma severity scores among the included studies exhibited inconsistencies, so data preprocessing was necessary before conducting comparisons, with review manager version 5.4 [9]. Effect sizes in the study were expressed as standardized mean differences (SMD), which were calculated using means and standard deviations across different time points [10]. In cases where the mean and standard deviation were not reported, alternative methods were employed to estimate these values. A positive SMD indicates an improvement in melasma severity, with higher SMD values suggesting a greater improvement from baseline. Risk ratio (RR) was utilized to assess adverse reactions, clinical effectiveness, and patient satisfaction [9].

Sufficient information should be extracted from the study to compute the correlation coefficient ρ between pre- and post-treatment measurements. Sensitivity analysis is to change the ρ-value and then perform meta-analysis to check the impact on the results [9, 11].Effect-size estimates were examined along with their corresponding 95% confidence intervals and *P* values. A significance level of *P *< 0.05 was considered indicative of a substantial effect, aligning with the original statistical significance criterion [9].

Heterogeneity between studies was evaluated using the χ^2^ test and *I*^2^ statistic. *I*^2^ values ≤ 25% indicated no heterogeneity, ≤ 50% indicated minimal heterogeneity, ≤ 75% indicated moderate heterogeneity, and > 75% indicated significant heterogeneity [12]. Statistical methods for meta-analysis included the fixed effect model and the random effect model. Homogeneity was indicated by *P *> 0.1 or *I*^2 ^< 50%, and a fixed effects model was employed in such cases. For *P* ≤ 0.1 or *I*^2 ^≥ 50%, indicating significant heterogeneity, a random effects model was utilized. Due to the limited statistical power of heterogeneity tests, particularly when the number of studies was small, we primarily relied on descriptive analyses to assess heterogeneity. Funnel plots were assessed to investigate the association between the magnitude of the influencing scale and study precision, as well as examining potential biases in our study [9].

## Results

### Study Characteristics

**Fig. 1:**
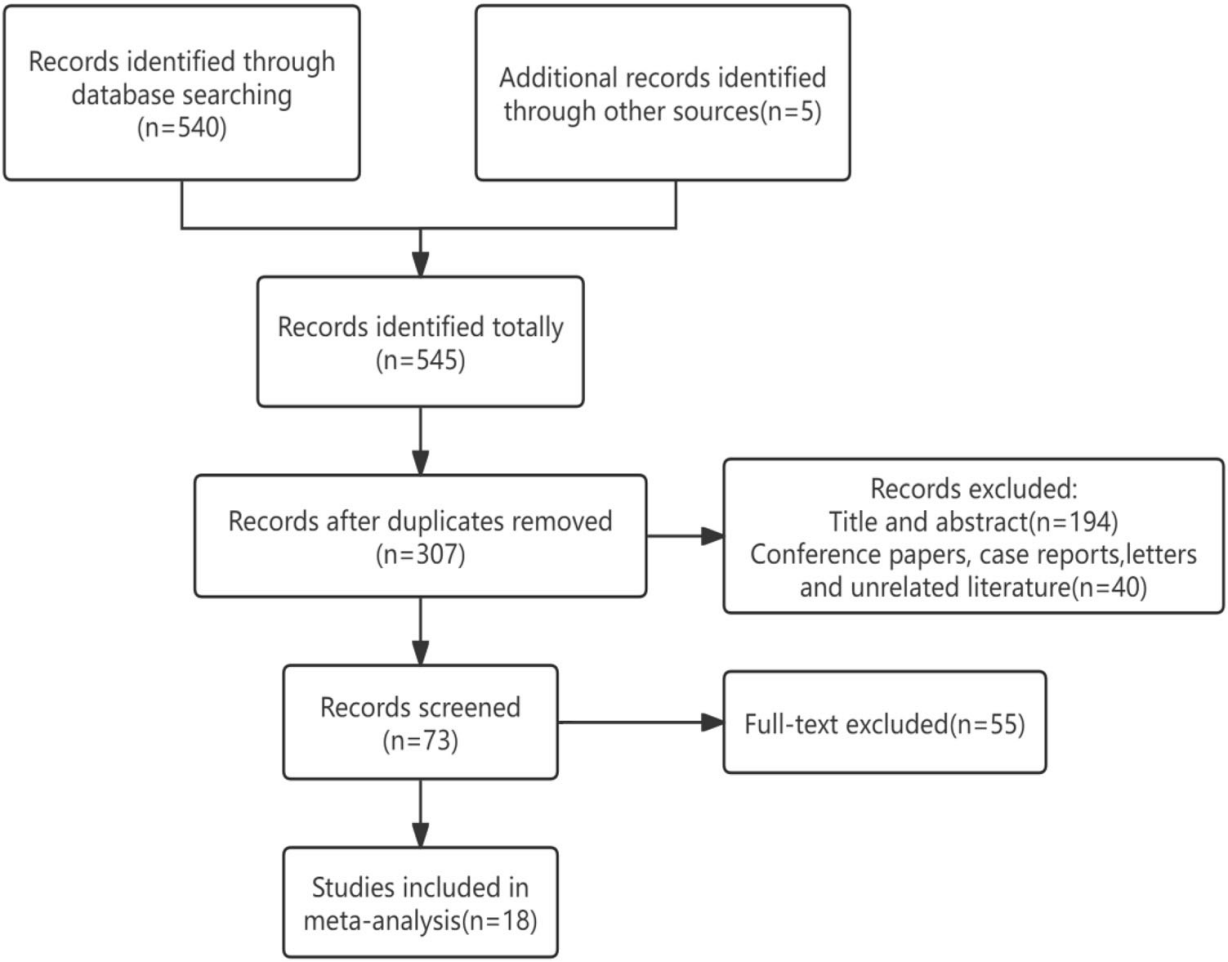
Procedure of literature screening

Using the search strategy described above, 545 citations were initially retrieved. After eliminating duplicate articles, reading titles and abstracts for preliminary screening, and then reading the full text, finally, a total of 18 articles [13–30] were included in this meta-analysis (Fig. 1). The 18 articles enrolled a total of 1245 patients, from 5 different countries, including China, Turkey, Egypt, Iran, Brazil, the proportion of female is relatively high, as high as 98.23%. Participants’ ages ranged from 18 to 60 years old and Fitzpatrick skin type ranged from I–V. Details of the 18 studies included in the analysis are shown in Table 1.

**Table 1:** Characteristics of included studies

Year, first author	Country	Study type	Quantity and gender	E/C	Age range (mean ± SD)	Fitzpatrick skin type	Microneedle characteristics	Dropout rate
2023, Poostiyan	Iran	RCT (split-face)	27 patients, (M: 0, F: 27)	27/27	(44.22 ± 8.39)	II–V	Microneedle; L = 2 mm	0%
2023, Fang Yuanfang	China	RCT	100 patients, (M: 0, F: 100)	50/50	25 ~ 47, (36.83 ± 4.83)	NA	Mechanical roller; L = 0.5 mm	0%
2022, Zhou Xiaoqin	China	RCT	140 patients, (M: 0, F: 140)	70/70	E: (36.74 ± 12.03); C: (39.92 ± 13.02)	III–IV	Mechanical roller	3.57% (reasons unspecified)
2022, Mao Aidi	China	RCT	140 patients, (M: 0, F: 140)	70/70	E: 25 ~ 52, (37.24 ± 6.38) C: 26 ~ 52,(36.58 ± 6.52)	NA	Mechanical roller	0%
2022, MA Wei	China	RCT	92 patients, (M: 0, F: 92)	30/31/31	E: (39.31 ± 8.23); C1: (37.32 ± 7.69) C2: (39.31 ± 8.23)	NA	Nanocrystalline	0%
2022, Hofny	Egypt	RCT	40 patients, (M: 0, F: 40)	20/20	NA	III–IV	Electrical repeating; L = 2.0 mm	0%
2021, Huang Linyu	China	RCT	92 patients, (M: 21, F: 71)	46/46	E: 18 ~ 54, (34.23 ± 10.45); C: 18 ~ 54, (34.20 ± 10.49)	III–IV	Mechanical roller L = 0.5 mm	0%
2021, Bergmann	Brazil	RCT	42 patients, (M: 6, F: 54)	21/21	18 ~ 50	I–IV	Mechanical roller microneedle; L = 1 mm	14.29% (personal reasons)
2020, Jing Juanjuan	China	RCT	66 patients, (M: 0, F: 66)	33/33	E: 31 ~ 49, (38.54 ± 5.01); C: 33 ~ 47, (38.31 ± 4.22)	NA	Mechanical roller L = 0.5 mm	0%
2020, Cassiano	Brazil	RCT	64 patients, (M: 0, F: 64)	16/16 /16/16	NA	NA	Mechanical roller L = 1.5 mm	1.6% (personal reasons)
2019, Zhang Yujie	China	RCT	30 patients, (M: 0, F: 30)	15/15	28 ~ 52, (38.6)	III	Mechanical roller L = 0.5 mm	0%
2019, Saleh	Egypt	RCT	42 patients, (M: 0, F: 42)	24/24	E: 25–54, (39.28 ± 7.16) C: 24 ~ 56, (40.47 ± 6.14)	III–IV	Electrical repeating needling; L = 1.5 mm	0%
2019, H uang Yahua	China	RCT	108 patients, (M: 0, F: 108)	54/54	E: 2 4 ~ 55,(35.52 ± 2.47) C: 23 ~ 52, (35.52 ± 2.47)	NA	Mechanical roller	0%
2018, Sun Hui	China	RCT	94 patients, (M: 0, F: 94)	32/62	24 ~ 54,(36.24 ± 2.34)	II–IV	Mechanical roller L = 0.5 mm	0%
2017, Zhou Yan	China	RCT	51 patients, (M: 0, F: 51)	27/24	E: 23 ~ 60(35. 26 ± 11. 08); C: 21 ~ 58 (37. 21 ± 9.70)	II–IV	Mechanical roller L = 0.5 mm	0%
2017, Ustuner	Turkey	RCT (split-face)	16 patients, (M: 1, F: 15)	16/16	30 ~ 62, (37.69 ± 8.24)	NA	Electrical repeating L = 1.5 mm	12.5% (poor response, PIH)
2016, Jang He	China	RCT	39 patients, (M: 2, F: 37)	13/13/13	31 ~ 53, (42.18 ± 3.97)	III	Mechanical roller L = 0.5 mm	0%
2013, Budamakuntla	India	RCT	60 patients, (M: 6, F: 54)	30/30	18 ~ 50	IV–V	Mechanical roller L = 1.5 mm	26.67% (unknown reasons)

According to these 18 articles, 13 studies used mechanical roller microneedle ,3 studies used electrical repeating needling,1 study used nanocrystalline microneedle and 1 study did not describe the type of microneedle, the length of needle ranged from 0.5 to 2.0 mm. Most studies reported microneedle treatment at 2-week or 4-week intervals for the melasma. The interventions, adverse effects and effectiveness of enrolled studies are presented in Table 2.

**Table 2:** Interventions and the results compared of the included studies

Year, First author	Experimental group	Control group	Evaluate time	Evaluation item	Adverse events	Efficacy
2023, Poostiyan	Microneedle and 100 mg/ml TXA^9 ^× 3 sessions (4-week interval) and apply hydroquinone 4% cream for a month and using sunscreens (SPF^8 ^≥ 30) one month before	Inject 100 mg/ml TXA (0.1 ml) × 3 sessions (4-week interval) and Apply hydroquinone 4% cream for a month and using sunscreens (SPF ≥ 30)one month before	12 week	mMASI	E^3^: Erythema 23, Scaling 25, Edema 19, PIH ^7^1C^2^: Erythema 4, Scaling 2, Edema 4	[Microneedle and TXA] versus [Inject TXA] 1. mMASI reduction at week 12: 54.24% versus 43.06%
2023, Fang Yuanfang	Microneedle × 12 sessions (2-week interval) and Oral TXA 250 mg (BID^1^ 3 month)	Oral TXA 250 mg (BID 3 month )	12 week	MASI, clinical efficacy	NA^5^	[Microneedle and Oral TXA] versus [Oral TXA] 1. MASI reduction at week 12: 46.27% versus 30.28% 2. Clinical effective rate: 96.0% versus 84.00%
2022, Zhou Xiaoqin	Microneedle and Danshen injection and IPL^4 ^× 3 sessions (4-week interval)	IPL × 3 ssessions (4-week interval)	12week	MASI, patient satisfaction, clinical efficacy	NA	[IPL and Microneedle and Danshen injection] versus [IPL] 1. MASI reduction at week 12: 54.26% versus 25.43% 2. Clinical effective rate: 95.70% versus 67.70% 3. Patient satisfaction rate: 100% versus 89.23%
2022, Mao Aidi	Microneedle and TXA × 6 sessions (4-week interval) and Q-switch intervaltched Nd: YAG^10^ 1064 nm laser × 10 s(2-week interval)	Q-switch Nd: YAG 1064 nm laser × 10 sessions(2-week interval)	10 week, 20 week, 32 week	MASI, patient satisfaction, clinical efficacy	NA	[Microneedle and TXA and Q-switched Nd: YAG 1064 nm laser] versus [Q-switched Nd: YAG 1064 nm laser] 1. MASI reduction at week 10: 36.56% versus 28.35%2.MASI reduction at week 20: 54.72% versus 41.97% 3. MASI reduction at week 32: 48.54% versus 28.72% 4. Clinical effective rate: 98.57% versus 92.86% 5. Patient satisfaction rate: 95.71% versus 85.71%
2022, Ma Wei	Microneedle and TXA × 6 sessions(4-week interval) and oral TXA 250 mg (BID 6 month)	1. Oral TXA 250 mg (BID 6 month) 2. Microneedle and TXA × 6 sessions (4-week interval)	24 week	MASI, patient satisfaction, clinical efficacy	E: Menstrual quantity decreases 1, Erythema 1, pain 1 C1:pain 1; C2: Erythema 2, Pain 2	[Microneedle and TXA and Oral TXA] versus [Oral TXA] versus [Microneedle and [TXA] 1. MASI reduction at week 24:72.53% versus 60.31%; 2. Patient satisfaction: 90% versus 67.74% versus 58.06% 3. Clinical effective rate: 96.67% versus 90.32% versus 90.32%
2022, Hofny	Microneedle × 4 sessions (4-week interval) and trichloroacetic acid × 8 sessions (2week interval)	Trichloroacetic acid × 8 sessions (2-week interval)	16 week, 24 week	MASI, patient satisfaction	E: burning 7, erythema 3, folliculitis 1; C: burning 9, erythema 8, PIH 1	[Microneedle and trichloroacetic acid] versus [trichloroacetic acid] 1. MASI reduction at week 16: 58.53% versus 48.3% 2. MASI eduction at week 24: 59.49% versus 48.3% 3. Patient satisfaction rate: 90% versus 75%
2021, Huang Linyu	Microneedle and apply a mixture of injection cosmetic repair enzyme I and lyophilized powder I and apply a compressed ice crystal protein mask × 3 sessions (4-week interval) and Oral TXA 250 mg (BID 3 month)	Oral TXA 250 mg (BID 3 month)	12 week	MASI, clinical efficacy	NA	[Microneedle and Humanlike collagen biorepair dressing and Oral TXA tablets] versus [Oral TXA tablets] 1. MASI reduction from baseline at week 12:43.92% versus 20.91% 2. Clinical effective rate: 95.65% versus 82.61%
2021, Bergmann	Microneedle and 5% retinoic acid × 5 sessions (15-day interval)	Retinoic acid treatment alone × 5 sessions (15-day interval)	4 week, 8 week	MASI	NA	[Microneedle and retinoic acid solution] versus [Retinoic acid solution] 1. MASI reduction at week 4: 32.99% versus 35.57% 2. MASI reduction at week 8 MASI: 46.39% versus 49.49% 3. Patient satisfaction 14: 66.6% versus 70.6%
2020, Jing Juanjuan	Microneedle × 12 sessions (2-week interval) and Oral TXA 250 mg(BID 6 month)	Oral TXA 250 mg (BID 6 month)	24 week	MASI, clinical efficacy	NA	[Microneedle and Oral TXA] versus [Oral TXA] 1. MASI reduction at week 24:54.58% versus 33.5% 2. Clinical effective rate: 90.90% versus 69.70%
2020, Cassiano	MT: microneedle × 2 sessions(1 month interval) and oral 250 mg TXA BID 2 month	①M: microneedle × 2 ssessions(1-month intervals) and oral placebo 250 mg BID 2 month ②T: oral TXA 250 mg BID 2 month ③CT: oral placebo 250 mg BID 2 month	4 week, 8 week, 16 week	mMASI	persistent headache 1, herpes simplex 3	[Oral placebo] versus Oral [TXA] versus [Microneedle and Oral placebo] versus [Microneedle and Oral TXA] 1. mMASI reduction at week 4: − 11.1% versus 38.0% versus 32.3% versus 59.3% 2. mMASI reduction at week 8:11.1% versus 64.0% versus 46.2% versus. 51.9% 3. mMASI reduction at week 16:19.4% versus 42.0% versus 47.7% versus 50.0%
2019, Zhang Yujie	Microneedle and Human-like collagen × 6 sessions (4-week interval) and Q-switch intervaltch 1064 nm laser × 6 sessions (4-week interval),alternately for 2 weeks	Q-switch 1064 nm laser × 6 ssessions (4-week interval)	24 week	MASI, patient satisfaction, clinical efficacy	NA	[Microneedle and Human-like collagen and Q-switch 1064 nm laser] versus [Q-switch 1064 nm laser] 1. MASI reduction at week 24: 62.45% versus 40.83% 2. Clinical effective rate: 93.33% versus 80.00% 3. Patient satisfaction rate :86.67% versus 73.33%
2019, Saleh	Microneedle and TXA × 6 ssessions (2-week interval)	Microneedle × 6 ssessions (2-week interval)	12 week	MASI	NA	[Microneedle and TXA] versus [Microneedle] 1. MASI reduction at week 12: 62.1% versus 22.5%
2019, Huang Yahua	Microneedle × 12 sessions (2-week interval) and Oral TXA 250 mg (BID 6 month)	Oral TXA 250 mg(BID 6 month)	8 week, 16 week, 24 week	MASI, clinical efficacy	E: laxativeness 6, menoxenia 6, PIH 2; C: laxativeness 5, menoxenia 7	[Microneedle and Oral TXA] versus [Oral TXA] 1. MASI reduction at week 8:23.58% versus 11.99% 2. MASI reduction at week 16: 39.45% versus 26.12% 3. MASI reduction at week 24:56.55% versus 40.03% 4. Clinical effective rate :94.44% versus 72.22%
2018, Sun Hui	Microneedle and TXA × 4 sessions and Q-switch 1064 nm laser × 6 sessions (2-week interval)	Q-switch 1064 nm laser × 6 sessions (2-week interval)	4 week, 8 week, 24 week	MASI, patient satisfaction	E: erythema 2; C: PIH 5, swelling 3, erythema 4	Microneedle and TXA and then perfromed Q-switch 1064 nm laser versus Q-switch 1064 nm laser 1. MASI reduction at week 4: 5.96% versus 3.17% 2. MASI reduction at week 8: 24.67% versus 13.25% 3. MASI reduction at week 24: 24.67% versus 13.25% 4. Patient satisfaction rate :81.25% versus 64.25%
2017, Zhou Yan	Microneedle and vitamin C × 5 sessions(2-week interval) and Human-like collagen and hydroquinone cream (BID)	Human-like collagen and hydroquinone cream (BID)	12 week	MASI, patient satisfaction, clinical efficacy	E: itching 6, erythema 6 C: itching 3	[Microneedle and vitamin C and Human-like collagen and Hydroquinone cream] versus [Human-like collagen and Hydroquinone cream] 1. MASI reduction at week 12:51.02% versus 26.03% 2. Clinical effective rate: 81.48% versus 50.00% 3. Patient satisfaction rate: 77.78% versus 54.17%
2017, Ustuner	Q-switch Nd:YAG laser and microneedling and vitamin C × 4 sessions(4-week interval)	Q-switch Nd:YAG laser × 4 sessions (4-week interval)	4 week, 8 week, 12 week, 16 week	MASI, patient satisfaction	E: erythema and PIH 1, Irritation 2, hypopigmenta-tion 2 C: erythema 2, PIH 2, irritation 1	[Q-switch Nd: YAG and microneetle and Vitamin C] versus [Q-switch Nd: YAG] 1. MASI reduction at week 4: 33.66% versus 3.59% 2. MASI reduction at week 8: 42.47% versus 8.48% 3. MASI reduction at week 12:57.1% versus 18.11% 4. MASI reduction at week 16: 64.63% vs. 26.26% 5. Patient satisfaction rate: 64.29% versus 14.28%
2016, Jang He	Microneedle × 3 sessions and OPT^6 ^× 3 sessions ,alternately for 4 weeks	1. Microneedle × 6 sessions (4-week interval) 2. OPT × 6 sessions(4-week interval)	36 week	MASI	E: skin dryness 4, erythema 1 OPT: erythema 2, PIH 1, skin dryness 9;	Microneedle and OPT versus. Microneedle versus OPT: 1.MASI reduction at week 36: 25.64% versus 13.75% versus 8.42%
2013, Budamaku-ntla	Microneedle and TXA × 3 sessions(4-week interval)	Microinjections of TXA × 3 sessions(4-week interval)	4 week, 8 week, 12 week, 16 week, 20 week	MASI	E: Itching 3, Burning 2, Erythema 4; C: Itching 3, Burning 2, Erythema 4	[Microneedle and TXA] versus. [Microinjections of TXA] 1. MASI reduction at week 4: 32.45% versus 18.39%; 2. MASI reduction at week 8: 40.59% versus 28.63%; 3. MASI reduction at week 12:42.71% versus 31.32%; 4. MASI reduction at week 16:44.41% versus 34.21%; 5. MASI reduction at week 20:44.41% versus 35.72%

1. BID, twice daily; 2. C, control group; 3. E, experimental group; 4. IPL, intense pulsed light; 5. NA, not available; 6. OPT, optimal pulse technology; 7. PIH, post-inflammatory hyperpigmentation; 8. SPF, sun protection factor; 9. TXA, tranexamic acid; 10. Nd:YAG, Neodymium-doped yttrium aluminium garnet.

### Quality Evaluation

The quality of each study included in this meta-analysis was evaluated according to the RoB 2.0 tool [8]. All included literatures mentioned random sequence generation; 15 studies mentioned allocation concealment; 9 studies mentioned blinding of subjects and participants; 8 studies used blinded outcome evaluators.

**Fig. 2:**
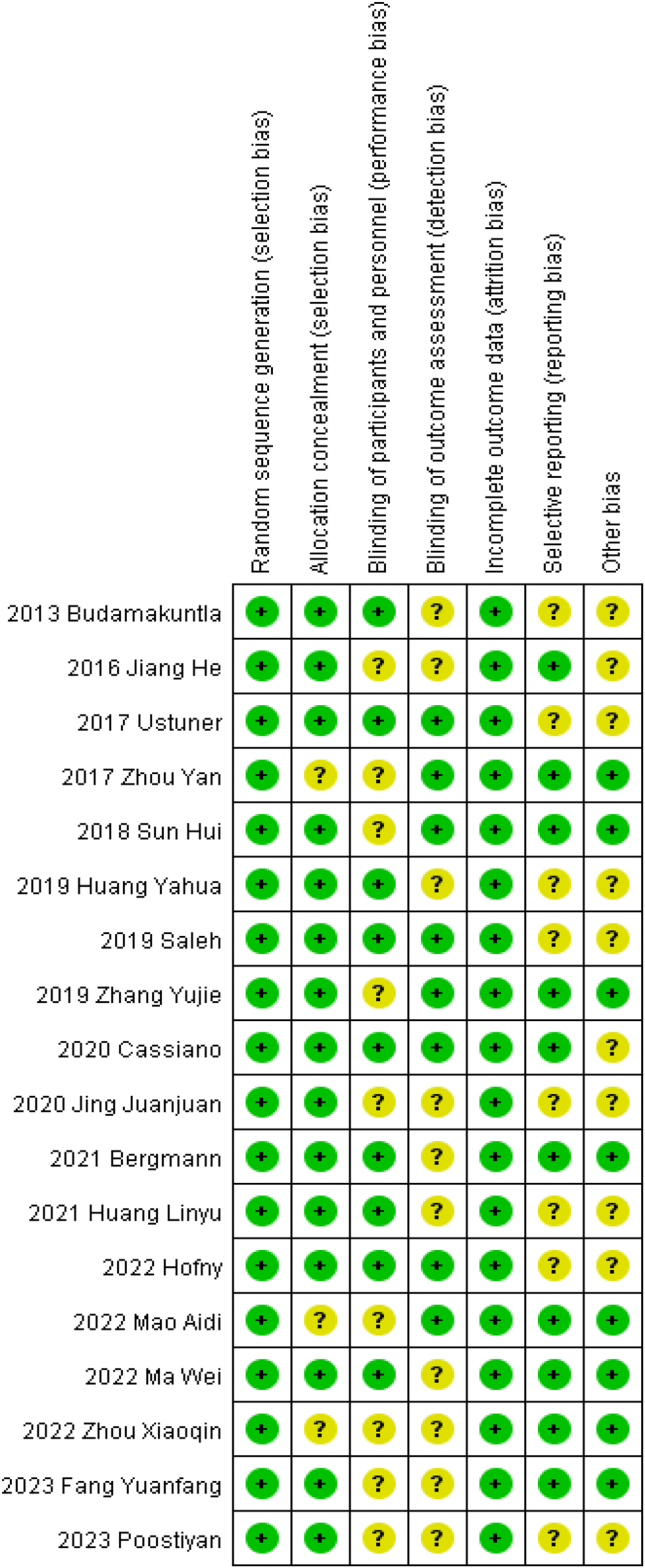
Bias risk assessment of included studies

All studies had complete data analysis; 9 had selective reporting of outcomes, and 10 had other possible sources of bias. The results of the bias risk assessment are presented in Fig. 2, which shows that the risk of bias in the included studies was low risk.

### Meta-Analysis

This meta-analysis compares: (1) variation of microneedle-assisted therapy of MASI from baseline to each timepoint; (2) comparison of local microneedle-assisted oral drug with oral drug alone; (3) the change of MASI in microneedle-assisted laser therapy and laser therapy alone; (4) comparison of microneedle-assisted therapy with microinjection therapy for TXA; (5) patient satisfaction; (6) clinical efficacy; (7) adverse events. A meta-analysis was performed only when 2 or more studies reported the same outcome. The meta-analysis results were interpreted according to published guidelines [31]. Literatures were analyzed descriptively if they did not provide sufficient data for inclusion in a meta-analysis.

Among the 18 studies, 2 studies reported mMASI scores and other 16 studies reported MASI scores. We used Cohen methods [10] to standardize the data and to assess the difference in melasma severity between the observation and control groups. The results of data analysis MASI before and after treatment of the measured value of the correlation ρ was selected as 0.6 [32]. We tried different ρ value and found that estimates of the effect of 95% CI is not sensitive to the choice of correlation. The results of a sensitivity analysis performed assuming no correlation between pre- and post-treatment measurements are reported in Supplemental Figure (S1 , 2, 3).

### Variation of Microneedle-Assisted Therapy of MASI from Baseline to Each Timepoint

**Fig. 3:**
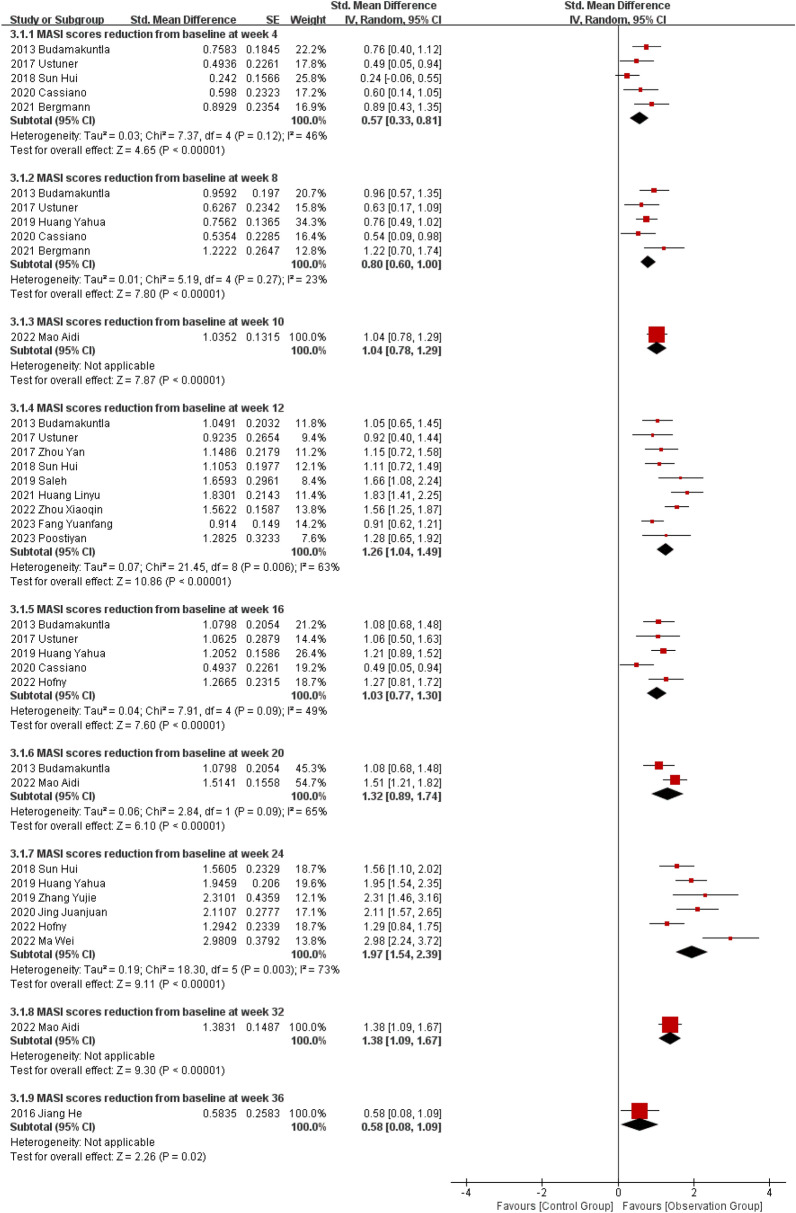
Forest plot illustrating the variation of MASI from baseline to each timepoint for microneedle-assisted therapy

The combined effect size of the data for included literatures demonstrated a positive outcome ultimately as shown in Fig. 3. These findings suggest that the microneedle-assisted therapy in the treatment of melasma has a significantly effective, meanwhile, as the treatment duration extended, the combined effect size exhibited greater magnitude and pronounced more efficacy, the difference was statistically significant. The effects at the 4th week (SMD = 0.57, 95% CI [0.33, 0.81], *P *< 0.00001), 8 weeks (SMD = 0.80, 95% CI [0.60, 1.00], *P *< 0.00001), the 12th week (SMD = 1.26, 95% CI [1.04, 1.49], *P *< 0.00001), the 16th week (SMD = 1.03, 95% CI [0.77, 1.30], *P *< 0.00001) and the 20th week (SMD = 1.32, 95% CI [0.89, 1.74], *P *< 0.00001) after treatment initiation. The most substantial reduction of MASI was observed at the 24th week. Furthermore, Mao’s [26]and Jiang’s[14] study mentioned the persistent reduction of MASI at the 32nd week (SMD = 1.38) and the 36th week (SMD = 0.58).

### Comparison of Local Microneedle-Assisted Oral Drug with Oral Drug Alone

Six studies [19, 21, 22, 24, 25, 30] recorded the data of MASI reduction in microneedle-assisted oral drug and oral drug alone. As shown in (S4), there was no significant difference between the microneedle-assisted oral drug group and oral drug group at the 8th week (SMD = 0.27, 95% CI [− 0.26, 0.80], *P* = 0.32) and 12 weeks (SMD = 1.72, 95% CI [− 0.04, 3.39], *P* = 0.06), until after 16 weeks (SMD = 0.44, 95% CI [0.11, 0.78], *P *= 0.01) both oral drug group and microneedle-assisted oral drug group have demonstrated efficacy in ameliorating the severity of melasma, while microneedles assisted with oral medication is more significant, and the significance remained at the 24th week(SMD = 0.55, 95% CI [0.15, 0.95], *P* = 0.007).

### Comparison Between Microneedle-Assisted Laser Therapy and Laser Therapy Alone

According to the data of 5 included studies [15, 17, 20, 26, 27], microneedle-assisted laser therapy has been demonstrated to be more effective than laser therapy alone. The analysis results in (S5) showed that MASI reduction has no significant difference between microneedle-assisted laser therapy and laser therapy alone at the 4th week (SMD = 0.22, 95% CI [− 0.15, 0.60], *P* = 0.24). While the significant difference has appeared at the 8th week (SMD = 0.58, 95% CI [0.20, 0.95], *P* = 0.003), and it was continued to be effective at the 24th week (SMD = 0.70, 95% CI [0.32, 1.08], *P* = 0.0003).

### Comparison of Microneedle-Assisted Local Therapy with Local Therapy Alone

Three studies [16, 23, 28] have demonstrated that microneedle-assisted therapy is more effective than local therapy for improving the severity of melasma. Zhou’s study [16] showed that microneedle-assisted therapy improved MASI scores twice as much as local therapy (MASI reduction: 51.02% vs. 26.03), the clinical efficiency (81.48% vs. 50%) and patient satisfaction (77.78% vs. 54.17%) were greatly improved meanwhile. Hofny’s study [28] reported that at the 16th week (MASI reduction: 58.53% vs. 48.3%), both groups produced significant curative effect and continued to the 24th week (MASI reduction: 59.49% vs. 48.3%), but the microneedle-assisted treatment group had better results and higher patient satisfaction (90% vs. 75%). Bergmann’s study [23] reported similar rates of reduction and patient satisfaction in the two groups, with higher rates of reduction at the 8th week (MASI reduction: 46.39% vs. 49.49%) than at the 4th week (MASI reduction: 32.99% vs. 35.57%).

### Comparison of Microneedle-Assisted Therapy with Microinjection Therapy for the Same Drug

The results of two studies [13, 29] demonstrated a significant reduction in the severity of melasma through microneedle-assisted therapy and microinjection therapy. Microneedling exhibited superior efficacy compared to microinjection in both studies at the 12th week (SMD = 0.58, 95% CI [0.13, 1.03], *P* = 0.01), meanwhile, the MASI reduction of microneedle-assisted therapy and microinjection therapy at 20 weeks in Budamakuntla’ study [13] achieved 44.41% and 35.72%, respectively.

### Comparison of Microneedle-Assisted Therapy with Microneedle Therapy Alone

Microneedle alone and microneedle-assisted therapy could both improve the severity of melasma. With two studies [14, 18] reporting 13.75–22.5% improvement in the severity of melasma after 3–6 months of microneedles treatment, while it also reported that microneedle-assisted therapy had higher effect than microneedle alone (MASI reduction in Jiang’ study: 26.54% vs. 13.75%, MASI reduction in Saleh’ study: 62.1% vs. 22.5%).

### Patient Satisfaction

**Fig. 4:**
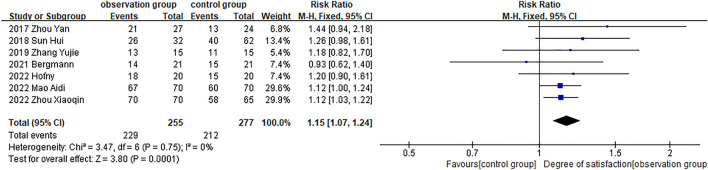
Forest plot for patient satisfaction

Patient satisfaction was reported in 7 studies [16, 17, 20, 23, 26–28], the overall combined effect of satisfaction was significant (RR = 1.15, 95% CI, [1.07, 1.24], *P* = 0.0001) in Fig. 4. Microneedle-assisted therapy had obviously higher patient satisfaction than the control group in 6 studies, while only Bergmann’s study[23] reported a 4% increase in satisfaction with microneedle-assisted topical TXA treatment compared to topical TXA alone (66.6% vs. 70.6%).

#### Clinical Efficacy

**Fig. 5:**
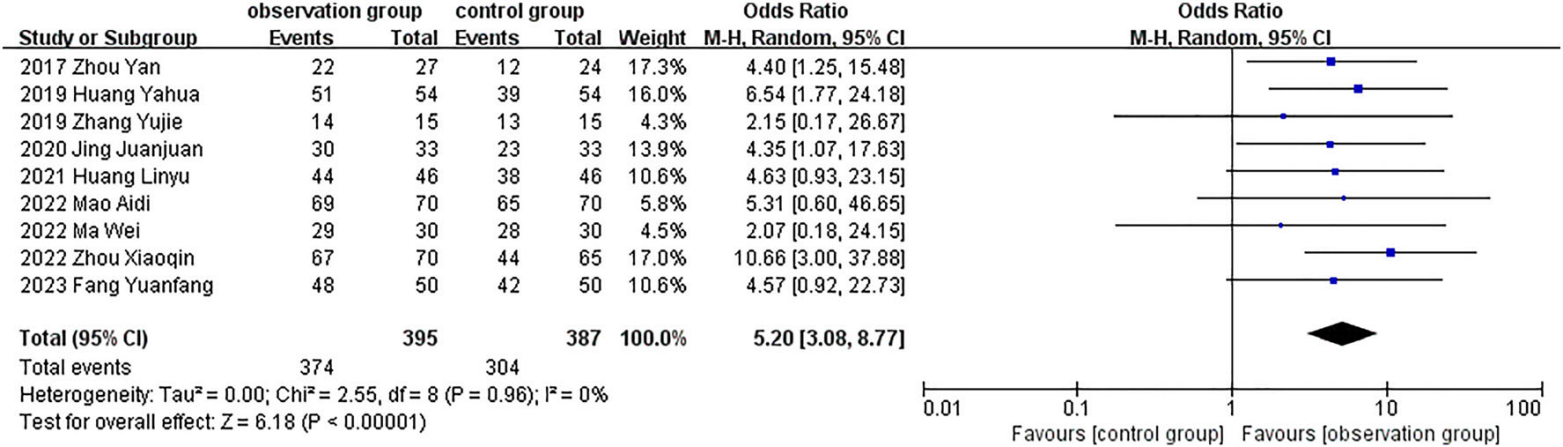
Forest plot for clinical efficacy

The clinical effective rate was defined as the total number of effective, improved and cured divided by the total number of treated patients. As shown in (Fig. 5), a total of 9 studies [16, 19, 20, 22, 24–27, 30] reported clinical effective rate, and the combined results indicated that microneedle adjuvant therapy was more significant than other monotherapy (RR = 1.18, 95% CI, [1.07, 1.30], *P* = 0.0007)

#### Adverse Events

Of all the reported adverse events to treatment regimens, microneedle was well tolerated. Common adverse reactions included erythema, itching, redness, and swelling as shown in (S6). The effect size of side effects and adverse events was not significant. In Huang’s [19] study, tranexamic acid resulted in 5 cases of diarrhea and 7 cases of menstrual irregularities in oral TXA group, 6 cases of diarrhea and menstrual irregularities in observation group. Ma’s study [25] also reported 1 case of menstrual irregularities in both oral TXA group and observation group. Cassiano’s study [21] reported 1 case of persistent headache and 3 cases of herpes simplex in oral TXA group, which subsided after oral acyclovir. Hofny’s study [28] reported 1 case of folliculitis in microneedle-assisted topical trichloroacetic group. There were no serious adverse events in all studies (Fig. 5).

#### Publication *Bias*

The publication bias evaluation of the included studies was completed using the RevMan5.4 funnel plot. It can be observed in (S7) that the funnel plot is essentially symmetric around the central axis, two studies appeared at the bottom of the funnel plot, indicating a potential risk of bias due to their small sample size. Although an asymmetric shape was observed, it did not necessarily imply publication bias. Considering factors such as experimental site, subjects, protocols, treatment instruments, drugs, and return visit time, biases were expected to occur. S8 displays a funnel plot from a meta-analysis showing the improvement over time in the severity of melasma in patients receiving microneedle-assisted laser treatment compared to those receiving laser treatment alone. Comparison of microneedle-assisted therapy with oral medicine alone is shown in (S9).

## Discussion

All included randomized trials show that microneedle is an effective adjuvant therapy for melasma treatment. After 4 weeks, the effect of microneedle-assisted treatment began to be effective, and it was optimal at the 24th week. Meanwhile, microneedle also has higher patient tolerability and safety, with a lower incidence of adverse events. Compared with oral medication, microneedle-assisted therapy began to be more effective at week 12 and continued by 24 weeks. It is important to note that high heterogeneity was observed at week 12 due to limited reported results from only two studies. Huang’s study [19], which used more local repair products, showed stronger results after 12 weeks than other studies. Compared with laser therapy, microneedle-assisted therapy also showed stronger efficacy. Starting at the 8th week, microneedle-assisted treatment was significantly more effective, reaching its peak at the 12th week. Finally, in the comparison of microneedle and microinjection therapy, microneedle has always been more significant than micro-injection. In addition, the results of this meta-analysis showed that the clinical effective rate and patient satisfaction of patients with microneedle-assisted treatment were higher than those of the control group. As for adverse events shown in (S6), the incidence of burning sensation, pruritus and PIH in the microneedle-assisted treatment group was lower than that in the control group, but it has no significant differences.

The number of patients included in each study, patient race, number of microneedle treatments, treatment interval, main treatment regimen, follow-up time, and individual patient differences may affect the results of meta-analysis. Subgroup analyses were performed according to study design as shown in (S10–S13). The results of subgroup analysis were consistent with the overall analysis, microneedle-assisted treatment group was more effective than the control group, China had a more significant effect (SMD = 1.61, 95% CI, [1.29, 1.93], *P* < 0.001), it is probably related to the skin type, high incidence rate in Aisa women. It should be noted that, we divided the microneedle-assisted treatments into differrnt subgroups according to the number of microneedle treatments as shown in (S12), and it can be seen that 3 sessions(SMD = 1.29, 95% CI, [0.88, 1.69], *P* < 0.001)  started to have a significant effect until the maximum effect was reached with 6 sessions (SMD = 2.02, 95% CI, [1.32, 2.72], *P *< 0.001).

Melasma is an acquired disorder of hyperpigmentation, the etiology and pathogenesis of melasma are very complex, genetic susceptibility, ultraviolet radiation, and changes in estrogen levels are the three major causes [2, 33, 34]. In recent years, increased melanin synthesis in skin lesions, vascular proliferation, oxidative stress damage, inflammatory response and skin barrier damage have also been proved to be involved in the occurrence of melasma [34, 35]. In addition, phototoxic drugs and psychological factors can also cause the occurrence and development of melasma [34]. The treatment of melasma aims to inhibit the activity of melanocytes, reduce the synthesis and transport of melanin, and promote the degradation and destruction of melanin [36, 37]. The current treatment methods mainly include sun protection, topical drugs, oral drugs, chemical exfoliation, and physical laser therapy [18, 22, 26, 29]. However, the treatment of melasma is still a clinical problem due to the poor efficacy and high recurrence rate.

Microneedle, also known as percutaneous collagen induction therapy, is a minimally invasive procedure involving the delivery of fine needles into the skin, commonly through stamping, needle rollers, or electric-powered pen [38]. The mechanism of action supporting the efficacy of microneedling in the treatment of melasma includes: (1) Active the damage-repair mechanisms in the skin, fibroblast proliferation, collagen remodeling, repair of dermal and basement membrane damage, and normalization of skin structure [36, 38, 39]. (2) Promote the transdermal clearance of melanin and reduces the release of melanotropic stimulation signals [2, 37, 40]. (3) Increase skin immunity, reduce the colonization of pigment-producing bacteria in the skin, and reduce the formation of pigmentation [41]. (4) Achieves efficient delivery of drugs in the skin and improves the absorption and utilization of drugs [3, 42]. Current studies have reported the effectiveness of microneedles as an adjuvant therapy for melasma. Most of these studies have shown that microneedles as an adjuvant therapy gets a better clinical improvement, fewer adverse reactions, lasting efficacy and low recurrence rate than single treatment for melasma. Ramírez-Oliveros [43] conducted clinical trials on refractory melasma using a combination of 4% hydroquinone microneedle and oral medications. During a five-month follow-up, a noticeable decrease in MASI scores and a marked improvement in quality of life were observed, with no recurrences after five months. Hofny [28] utilized microneedle with trichloroacetic acid in the treatment of melasma, the result indicated that After 1 and 3 months of treatment, the mean melasma area and severity index, modified melasma area and severity index, and melasma severity index scores showed significant improvement in each group (*P *< 0.05 for each).

In summary, the research cited in this article outlines various treatments for melasma, with patient responses varying. However, based on subjective efficacy evaluations, microneedle appears to be a promising adjunctive treatment for melasma. This suggests that microneedle could have significant potential in future treatment plans for melasma. It may be particularly beneficial for patients who are unresponsive to single treatment modalities and can help maintain results in patients who have already achieved some level of efficacy. To achieve optimal results, combined treatments are generally necessary. Despite the relatively low incidence of adverse events with microneedle, physicians need to closely monitor patients and provide appropriate care. As microneedle is a novel concept, further high-quality randomized controlled trials are needed to determine the various combinations of microneedle with primary treatments and to develop more rational treatment strategies. In melasma treatment, alongside restoring normal skin color, attention should be given to the quality of the skin. Providing more personalized combined treatments for patients aiming to improve both melasma and skin quality will be valuable.

This study also has a few limitations. First, there were some differences in the design of the interventions of the included studies, but the main conclusions did not change when the study data were pooled according to the designed grouping. Second, due to the limited direct comparison of microneedle type, length, penetration depth, etc., in the included literature, the efficacy of microneedle in the treatment of melasma remains unclear. However, this article provides the best summary and explanation of the available evidence. Third, moderate heterogeneity was suggested in the meta-analysis of MASI improvement over time in microneedle-assisted therapy for melasma, but this heterogeneity is to be expected given the different topical therapies, treatment regimens, microneedle devices, and patient populations in the study. Lastly, the limited number of included studies led to the analysis of some indicators including only two to three articles. More relevant clinical trials should be conducted in the future. To further expand the sample size to verify the efficacy and safety of microneedle-assisted therapy.

In conclusion, this meta-analysis shows that microneedle-assisted therapy is an effective method of melasma treatment, the microneedle combination therapy provided significantly better outcomes than a single treatment. After 4 weeks, the effect of microneedle-assisted treatment began to be effective, it was optimal at 24 weeks, and with a high patient satisfaction and mild adverse effects. It provides encouraging evidence for the choice of clinical treatment for patients with melasma, clinicians can consider adding microneedling as a new option.

## Supplementary Information

Below is the link to the electronic supplementary material.

Supplementary file1 (DOCX 431 KB)

## References

[1] Zhou LL, Baibergenova A (2017) Melasma: systematic review of the systemic treatments. Int J Dermatol 56(9):902–908 28239840 10.1111/ijd.13578

[2] Passeron T, Picardo M (2018) Melasma, a photoaging disorder. Pigment Cell Melanoma Res 31(4):461–465 29285880 10.1111/pcmr.12684

[3] Qu F et al (2022) Advanced nanocarrier- and microneedle-based transdermal drug delivery strategies for skin diseases treatment. Theranostics 12(7):3372–3406 35547773 10.7150/thno.69999 PMC9065205

[4] Lee JC, Daniels MA, Roth MZ (2016) Mesotherapy, microneedling, and chemical peels. Clin Plast Surg 43(3):583–595 27363773 10.1016/j.cps.2016.03.004

[5] Moher D et al (2009) Preferred reporting items for systematic reviews and meta-analyses: the PRISMA statement. PLoS Med 6(7):e1000097 19621072 10.1371/journal.pmed.1000097 PMC2707599

[6] Silpa-Archa N et al (2015) Automated melasma area and severity index scoring. Br J Dermatol 172(6):1476 26036153 10.1111/bjd.13840

[7] Rodrigues M et al (2016) Interpretability of the modified melasma area and severity index (mMASI). JAMA Dermatol 152(9):1051–1052 27144383 10.1001/jamadermatol.2016.1006

[8] Sterne JAC et al (2019) RoB 2: a revised tool for assessing risk of bias in randomised trials. BMJ 366:l4898 31462531 10.1136/bmj.l4898

[9] Higgins JP Green S (2008) Cochrane handbook for systematic reviews of interventions

[10] Cohen J (1992) Statistical power analysis. Curr Dir Psychol Sci 1(3):98–101

[11] Borenstein M (2009) Effect sizes based on means. Introduction to meta-analysis, pp 21–32

[12] Hedges LV et al (2011) Introduction to meta-analysis. Wiley, New York

[13] Budamakuntla L et al (2013) A Randomised, open-label, comparative study of tranexamic acid microinjections and tranexamic acid with microneedling in patients with melasma. J Cutan Aesthet Surg 6(3):139–143 24163529 10.4103/0974-2077.118403 PMC3800287

[14] He J (2016) Observation on the curative effect of novel intense pulsed light combined with skin needle rolling in the treatment of melasma. Chin Med Cosmetol 6(11):51–54.

[15] Ustuner P, Balevi A, Ozdemir M (2017) A split-face, investigator-blinded comparative study on the efficacy and safety of Q-switched Nd:YAG laser plus microneedling with vitamin C versus Q-switched Nd:YAG laser for the treatment of recalcitrant melasma. J Cosmet Laser Ther 19(7):383–390 28657378 10.1080/14764172.2017.1342036

[16] Zhou Yan WL, Su F (2017) Clinical observation of 27 cases of melasma treated with microneedle roller combined with topical drugs. Chin J Dermatol Venereol 31(5):573–575

[17] Sun Hui WZ, Ni X (2018) Clinical observation of microneedle importing tranexamic acid into skin combined with Q switch 1 064 nm laser For treating chloasma. Chin J Derm Venereol 27(5):26–29

[18] Saleh F et al (2019) Topical tranexamic acid with microneedling versus microneedling alone in treatment of melasma: clinical, histopathologic, and immunohistochemical study. J Egypt Womens Dermatol Soc 16(2):89–96

[19] Huang Yahua YX (2019) Clinical efficacy of tranexamic acid combined with microneedle acupuncture in treating chloasma. Jiangsu Med J 45(10):1044–1047

[20] Zhang Yujie LY, Chen Y (2019) Observation on clinical efficacy of microneedle importing human-like collagen into skin combined with Q-switch 1 064 nm Nd:YAG laser in the treatment of melasma. Chin J Aesthet Med 28(5):8–10

[21] Cassiano D et al (2020) Efficacy and safety of microneedling and oral tranexamic acid in the treatment of facial melasma in women: an open, evaluator-blinded, randomized clinical trial. J Am Acad Dermatol 83(4):1176–1178 32035945 10.1016/j.jaad.2020.02.002

[22] Juanjuan J (2020) Clinical efficacy of microneedles combined with tranexamic acid in the treatment of melasma. J Med Aesthet Cosmetol 29(19):11–12

[23] Bergmann CLMS et al (2021) The use of retinoic acid in association with microneedling in the treatment of epidermal melasma: efficacy and oxidative stress parameters. Arch Dermatolo Res 313(8):695–704 10.1007/s00403-020-02140-8 32978675

[24] Huang Linyu CW (2021) Clinical study of microneedle repair combined with tranexamic acid tablets for chloasma. Shenzhen J Integr Tradit Chin Western Med 31(21):34–36

[25] Hua Gang MW Feng Y (2022) Observation on the efficacy of sodium crystal microneedle introduction combined with oral administration of tranexamic acid in the treatment of chloasma. Chinese Science and Technology Journal Database (Citation Edition) Medicine and Health (12)

[26] Yin Rui MA, Liao A (2022) Observation on the effect of Q-switched Nd: YAG 1064 nm laser combined with methothrapy on chloasma. Chin J Aesthetic Med 31(11):38–41

[27] Zhou Xiaoqin SM (2022) Analysis of therapeutic effect of salvia miltiorrhiza injection with intense pulse light combined with microneedles in the treatment of melasma. Mod Med Health 38(22):3911–3914

[28] Hofny ER et al (2023) Trichloroacetic acid with microneedling versus trichloroacetic acid alone for treating melasma. Dermatol Surg 49(1):66–71 36533799 10.1097/DSS.0000000000003641

[29] Poostiyan N et al (2023) Tranexamic acid microinjections versus tranexamic acid mesoneedling in the treatment of facial melasma: a randomized assessor-blind split-face controlled trial. J Cosmet Dermatol 22(4):1238–1244 36606390 10.1111/jocd.15580

[30] Fang Yuanfang LM (2023) An observation for clinical efficacy of derma roller combined with tranexamic acid in the treatment of chloasma. J Youjiang Med Univ Nationalities 45(05):778–781

[31] Treadwell JR et al (2006) A system for rating the stability and strength of medical evidence. BMC Med Res Methodol 6:52 17052350 10.1186/1471-2288-6-52 PMC1624842

[32] Bailey AJM et al (2022) Microneedling as an adjuvant to topical therapies for melasma: a systematic review and meta-analysis. J Am Acad Dermatol 86(4):797–810 33857549 10.1016/j.jaad.2021.03.116

[33] Sheth VM, Pandya AG (2011) Melasma: a comprehensive update: part I. J Am Acad Dermatol 65(4):689–697 21920241 10.1016/j.jaad.2010.12.046

[34] Basit H, KV Godse AM, Aboud Al (2023) Melasma. In: StatPearls, Treasure Island, FL

[35] Liu W, Chen Q, Xia Y (2023) New Mechanistic Insights of Melasma. Clin Cosmet Investig Dermatol 16:429–442 36817641 10.2147/CCID.S396272 PMC9936885

[36] McKesey J, Tovar-Garza A, Pandya AG (2020) Melasma treatment: an evidence-based review. Am J Clin Dermatol 21(2):173–225 31802394 10.1007/s40257-019-00488-w

[37] Lima EVA et al (2017) Assessment of the effects of skin microneedling as adjuvant therapy for facial melasma: a pilot study. BMC Dermatol 17(1):14 29183309 10.1186/s12895-017-0066-5 PMC5706369

[38] Wu SZ, Muddasani S, Alam M (2020) A systematic review of the efficacy and safety of microneedling in the treatment of melasma. Dermatol Surg 46(12):1636–1641 32897944 10.1097/DSS.0000000000002763

[39] Kim M et al (2019) Senescent fibroblasts in melasma pathophysiology. Exp Dermatol 28(6):719–722 30575141 10.1111/exd.13814

[40] Kang HY et al (2011) Transcriptional profiling shows altered expression of wnt pathway- and lipid metabolism-related genes as well as melanogenesis-related genes in melasma. J Invest Dermatol 131(8):1692–1700 21562572 10.1038/jid.2011.109

[41] Amani H et al (2021) Microneedles for painless transdermal immunotherapeutic applications. J Control Release 330:185–217 33340568 10.1016/j.jconrel.2020.12.019

[42] Cohen BE, Elbuluk N (2016) Microneedling in skin of color: a review of uses and efficacy. J Am Acad Dermatol 74(2):348–355 26549251 10.1016/j.jaad.2015.09.024

[43] Ramírez-Oliveros JF et al (2020) Microneedling with drug delivery (hydroquinone 4% serum) as an adjuvant therapy for recalcitrant melasma. Skinmed 18(1):38–40 32167455

