# Correction: Efficacy of Microneedle as an Assisted Therapy for Melasma: A Meta-analysis and Systematic Review of Randomized Controlled Trials

**DOI:** 10.1007/s00266-024-04544-7

**Published:** 2024-11-18

**Authors:** He Simin, Xue Siliang, Chen Wei, Diao Ping, Li Erlong, Zhao Jianbo

**Affiliations:** 1 Department of Dermatology, West China Hospital, Sichuan University, 37 Guoxue Lane, Wuhou District, Chengdu, 610041 Sichuan Province China; 2 Laboratory of Dermatology, Clinical Institute of Inflammation and Immunology, Frontiers Science Center for Disease-Related Molecular Network, West China Hospital, Sichuan University, Chengdu, 610041 China; 3 College of Information and Communication Engineering, Harbin Engineering University, Harbin, China

**Correction to: Aesthetic Plastic Surgery** 10.1007/s00266-024-04395-2

The original online version of this article was revised to add the following Funding notice:

This work was supported by the Sichuan Science and Technology Program (2023YFG0227).

The original article has been corrected.

