# Supplementary material for: Efficacy of Microneedle as an Assisted Therapy for Melasma: A Meta-analysis and Systematic Review of Randomized Controlled Trials
Source: Aesthetic Plast Surg. 2024 Oct 16;49(6):1755–69. doi: 10.1007/s00266-024-04395-2 (PMC11968554; doi:10.1007/s00266-024-04395-2)
Supplement: Supplementary file 1 — Supplementary file1 (DOCX 431 KB) [file 266_2024_4395_MOESM1_ESM.docx]

**Appendix:**


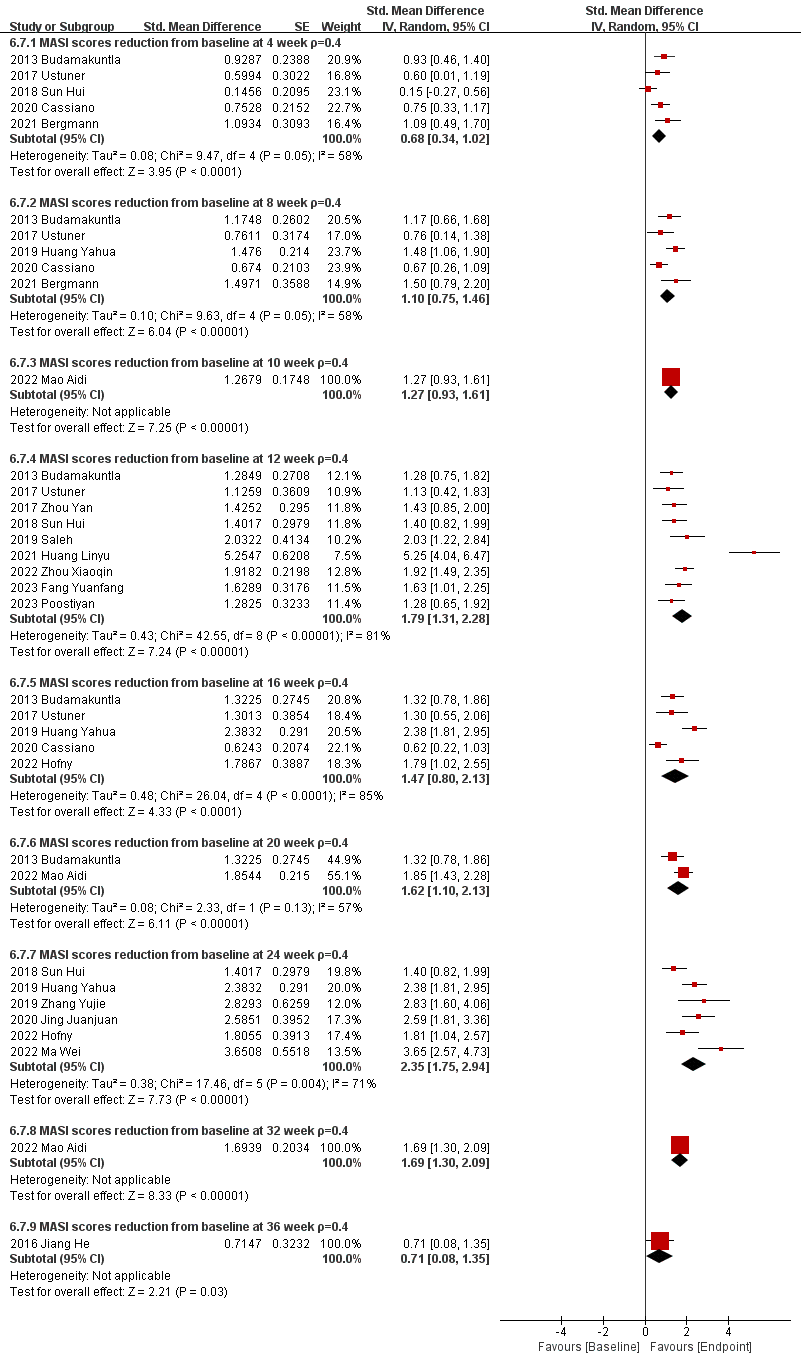


**S.1** Sensitivity analysis: The comparison of the reduction of melasma severity versus baseline over time in the assisted treatment with microneedles，ρ =0.4


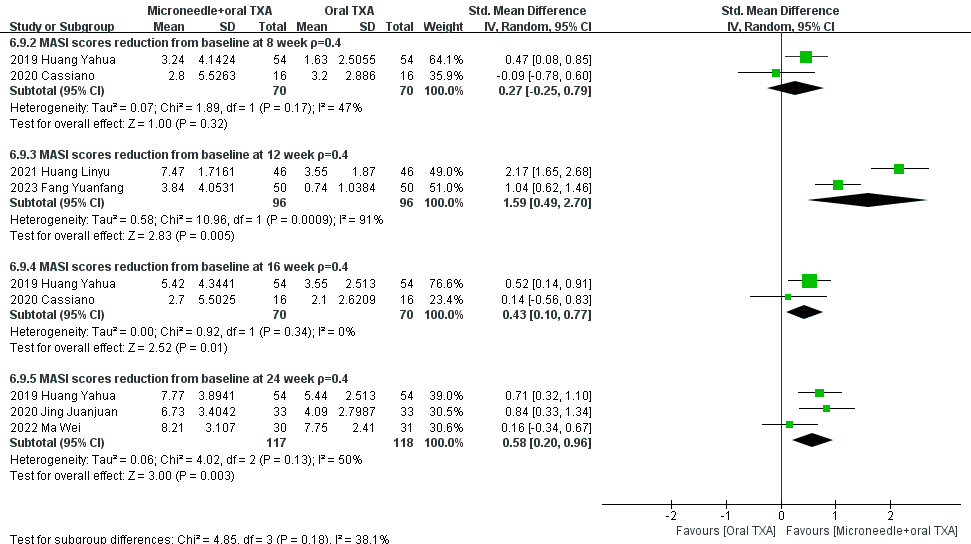


**S.2** Sensitivity analysis: The comparison of the reduction of melasma severity in microneedle-assisted oral drugs versus oral drugs，ρ =0.4


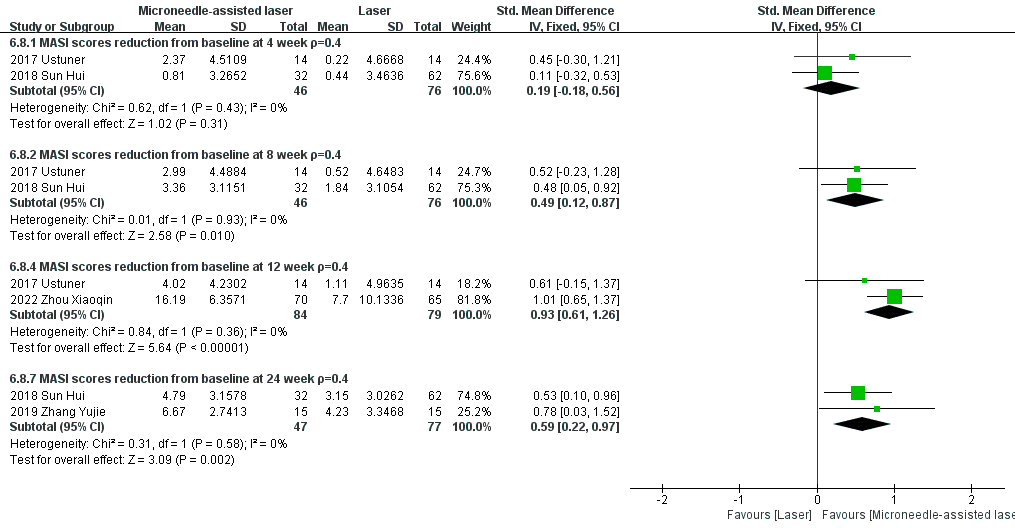


**S.3** Sensitivity analysis: The comparison of the reduction of melasma severity in microneedle-assisted laser versus laser，ρ =0.4

**
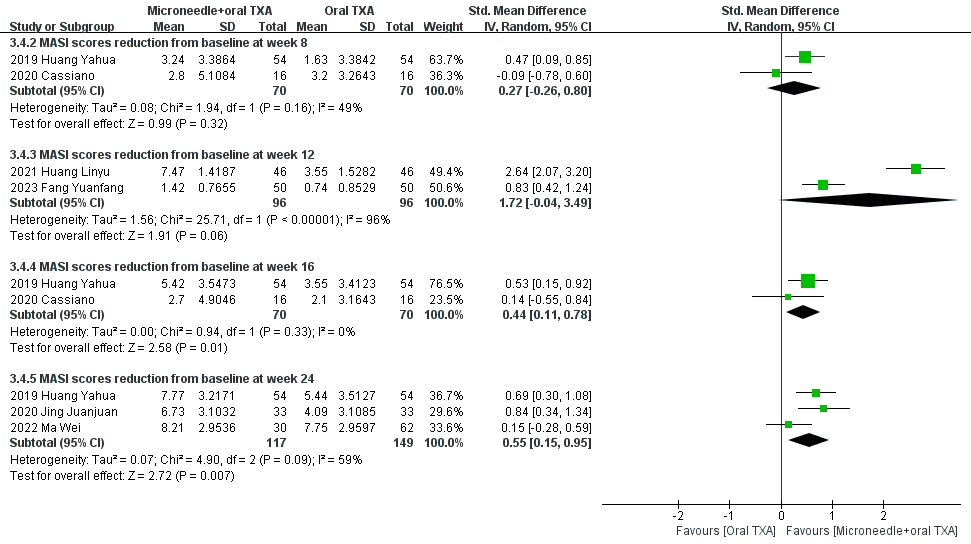
S.4** Forest plot showing the comparison of variation of MASI from baseline to each timepoint for local microneedle-assisted oral drug and oral drug alone


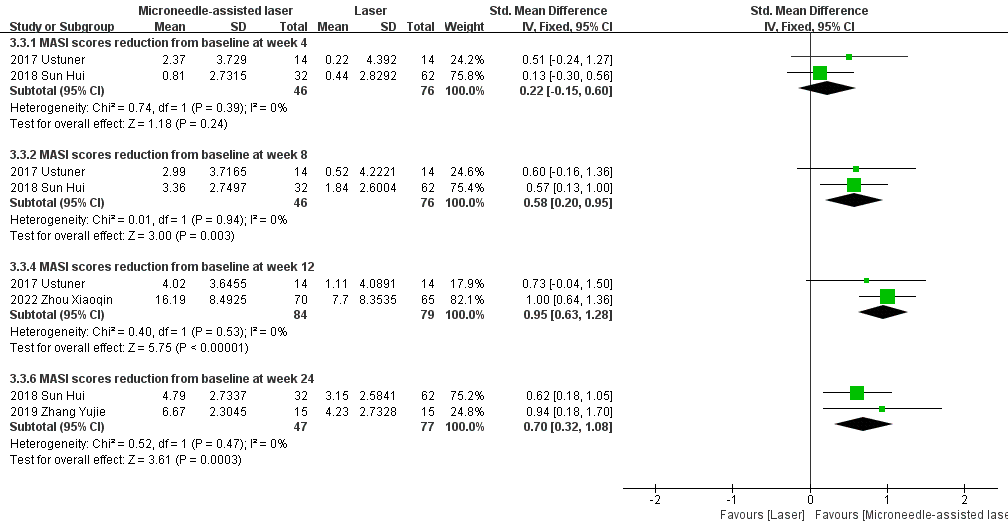


**S.5** Forest plot showing the comparison of variation of MASI from baseline to each timepoint for microneedle-assisted laser therapy and laser therapy alone


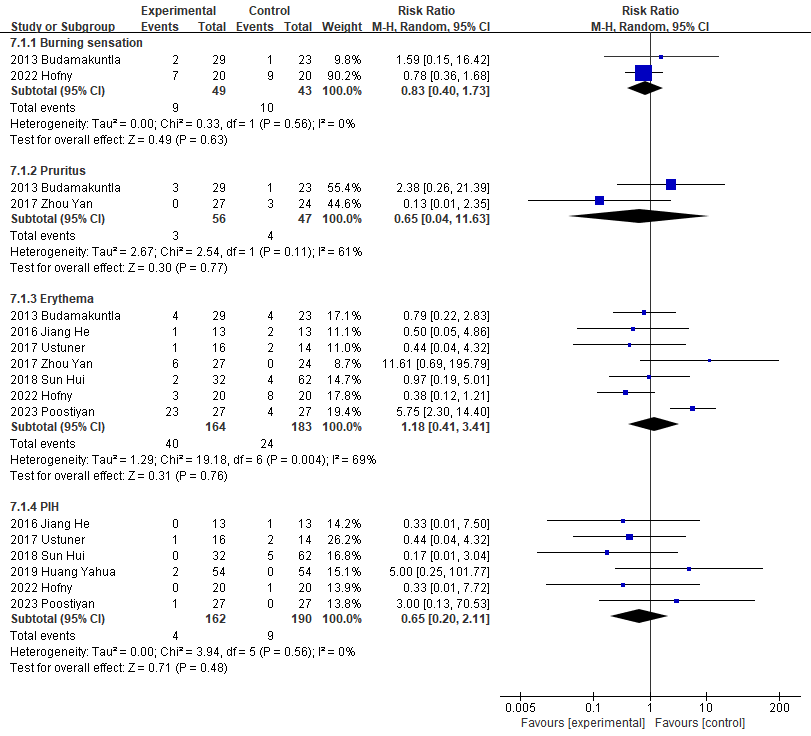


**S.****6** Forest plot for adverse events


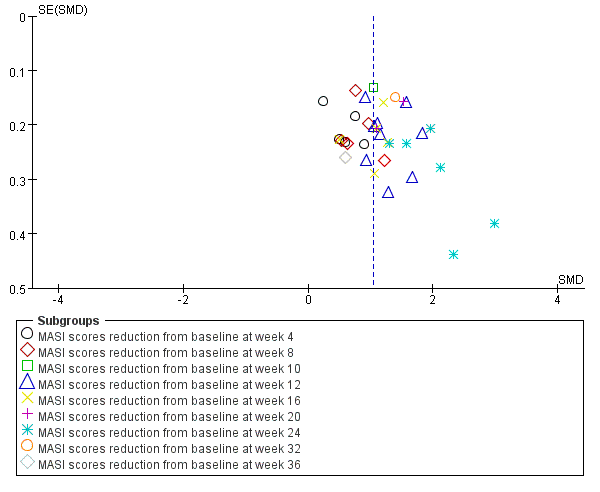


**S.7** Funnel plot for reduction in MASI from baseline to each timepoint for microneedle-assisted therapy


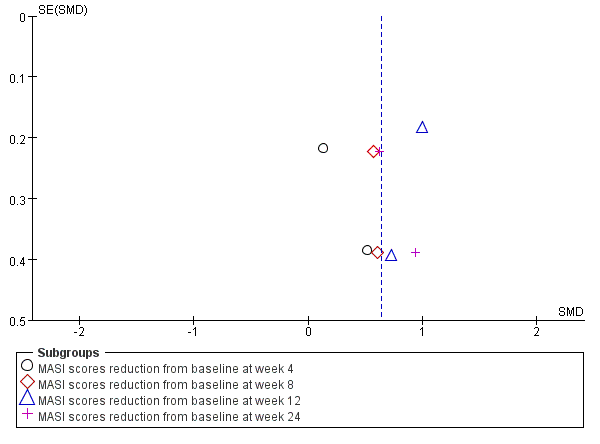


**S.8** Funnel plot for reduction in MASI over time of patients of microneedle-assisted laser therapy versus laser therapy alone


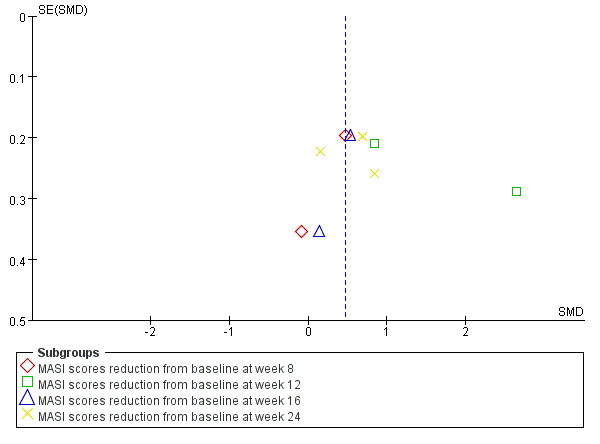


**S.9** Funnel plot for reduction in MASI over time of patients of microneedle-assisted oral drug with oral drug alone


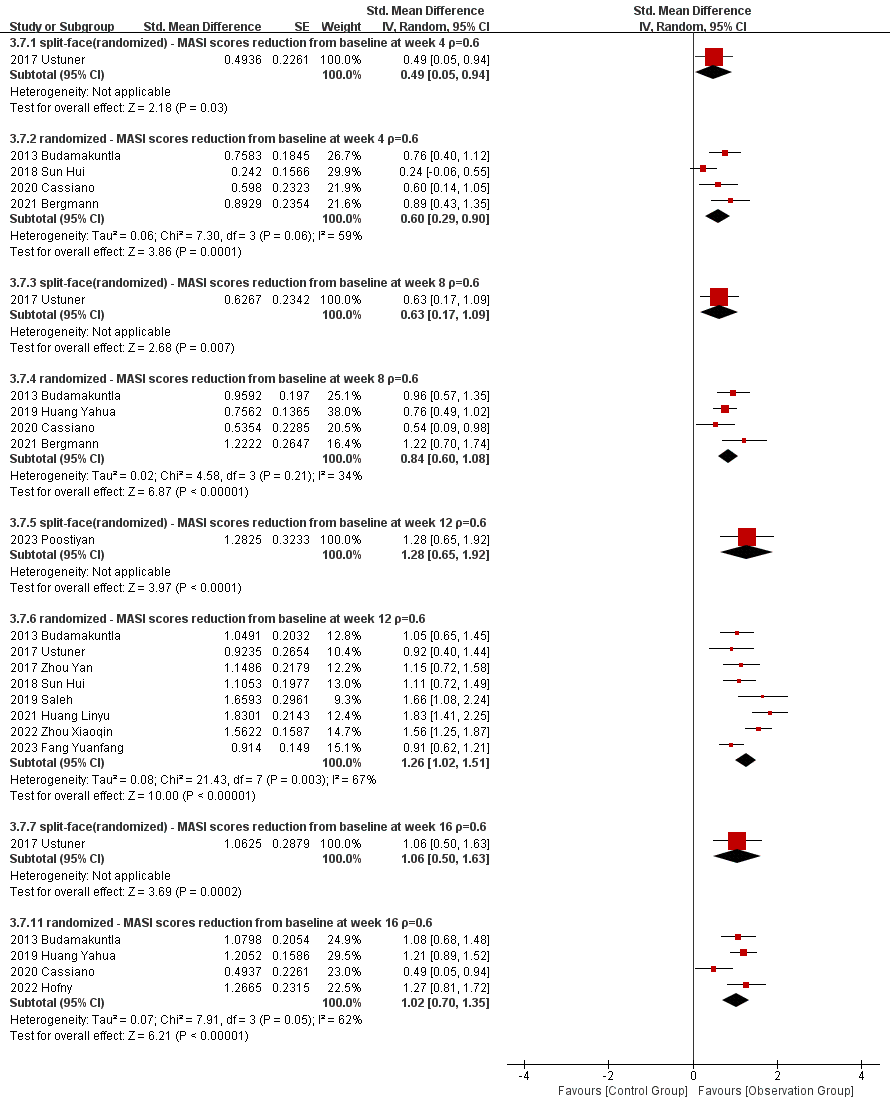


**S.10** Subgroup analysis: reduction in melasma severity from baseline in patients receiving microneedling-assisted treatment according to study type (randomized controlled, randomized split-face).


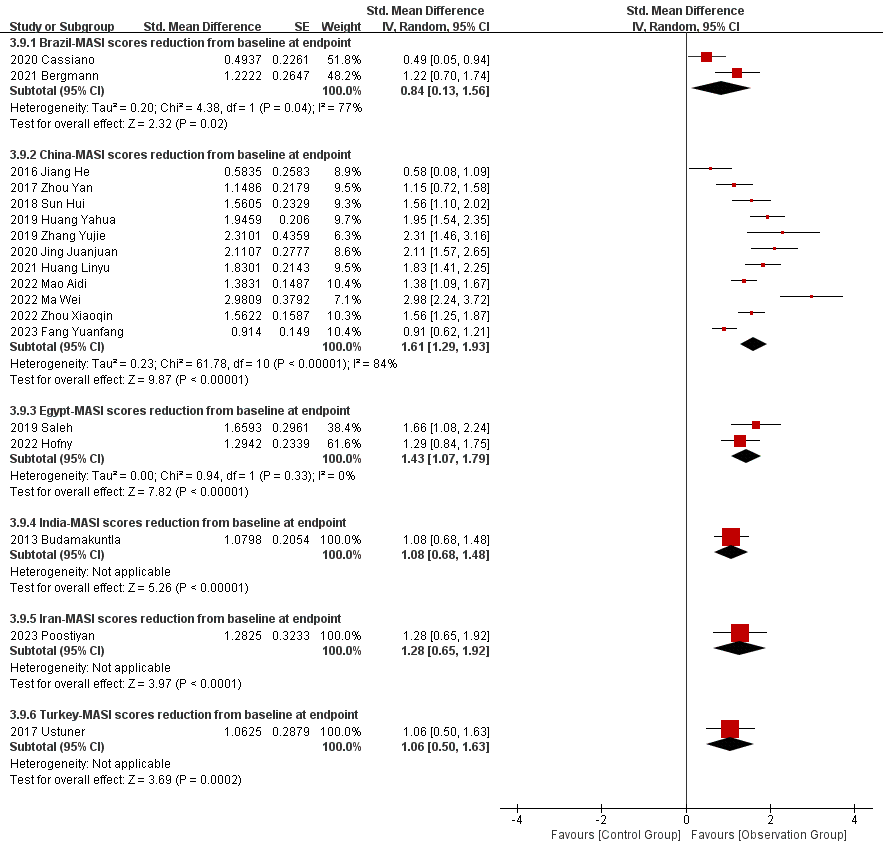


**S.11** Subgroup analysis: reduction in melasma severity from baseline in patients receiving microneedling-assisted treatment according to country. (Brazil, China, Egypt, India, Iran,Turkey)


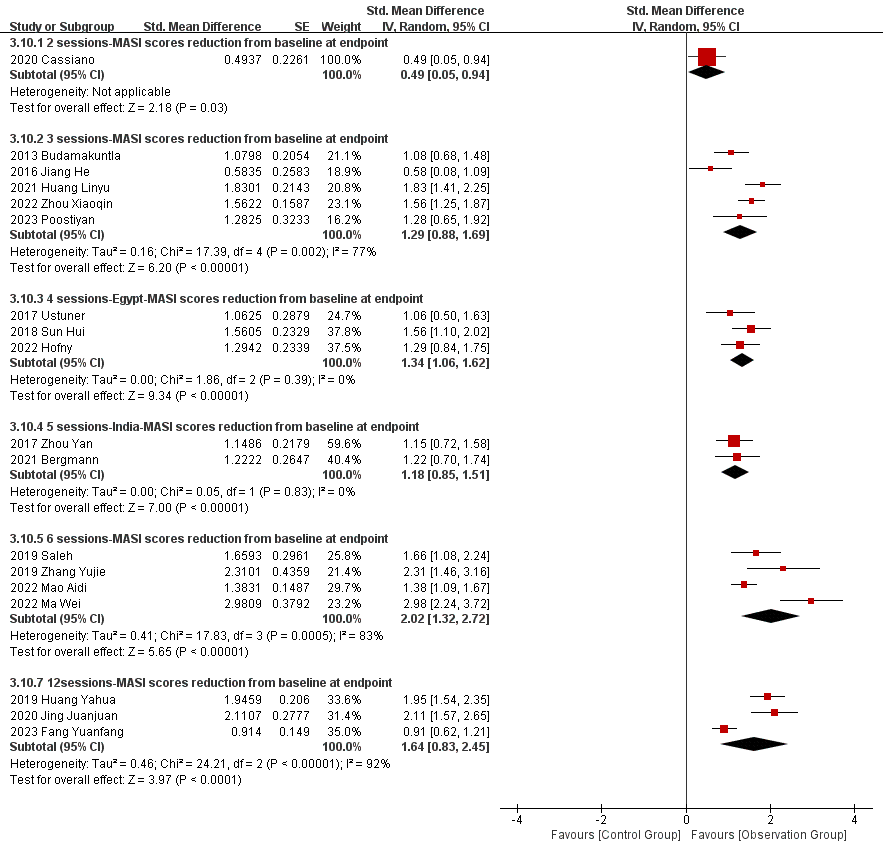


**S.12** Subgroup analysis: reduction in melasma severity from baseline in patients receiving microneedling-assisted treatment according to microneedling treatment times (2,3,4,5,6,12 sessions).


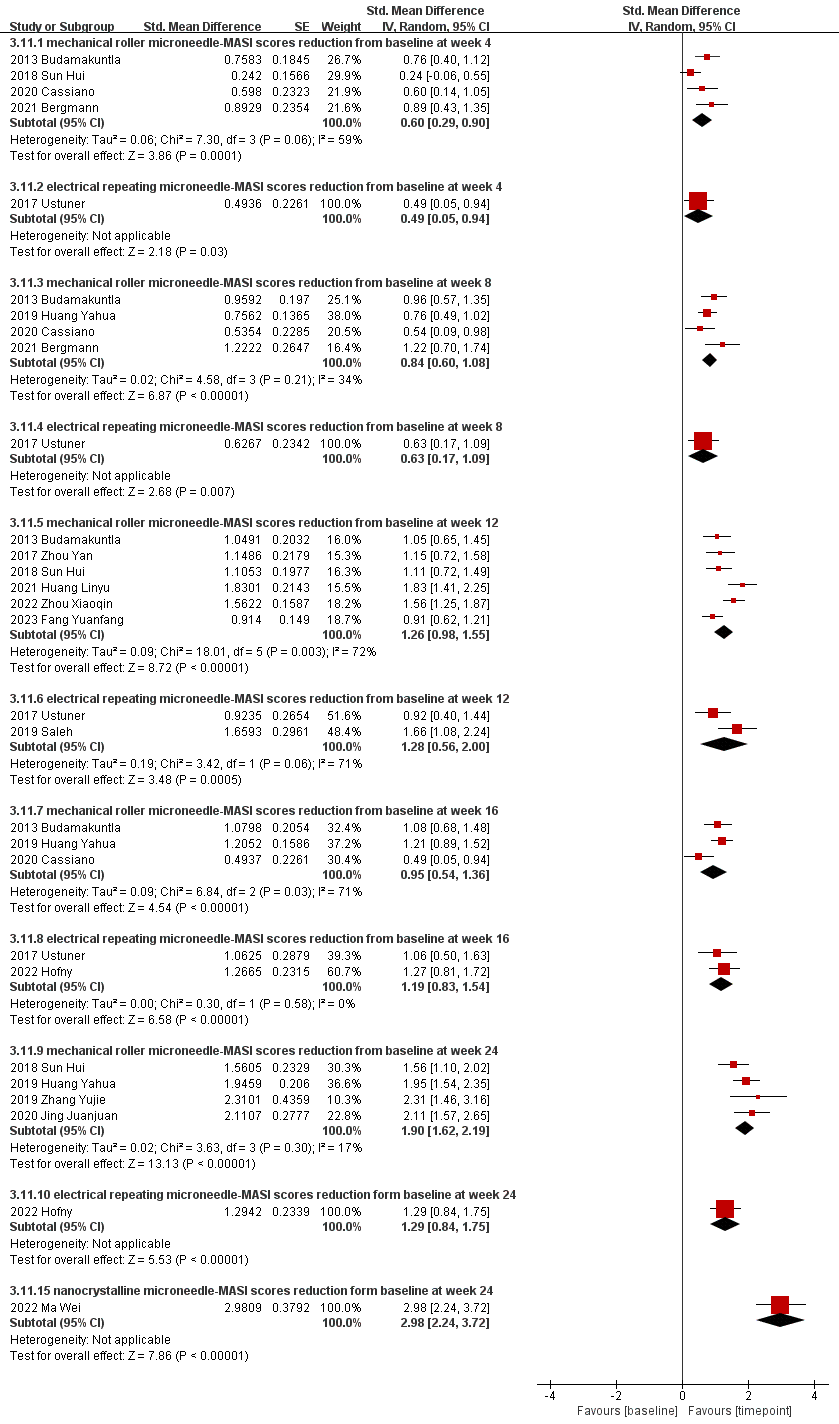


**S.13** Subgroup analysis: reduction in melasma severity from baseline in patients receiving microneedling-assisted treatment according to microneedle types(mechanical, electrical, nanocrystalline microneedles).

**Data Preprocessing**

**Within Group:Supplemental Methods for the Calculation of SMD**

The change in the Standardized Mean Difference (SMD) from baseline to endpoint within the same treatment group can be calculated using the following formula. Assuming a correlation coefficient $\rho$ between measurements.

$$d=\frac{MD}{SD}=\frac{{(Y}_{0}-Y_{1})\sqrt{2(1-\rho)}}{\sqrt{{(SD}_{0}^{2}-{SD}_{1}^{2})/2}}$$

${MD=Y}_{0}-Y_{1}$ represents the difference of MASI between the baseline mean and the endpoint mean.

$$SD=\frac{\sqrt{2(1-\rho)}}{\sqrt{{(SD}_{0}^{2}-{SD}_{1}^{2})/2}}$$

${SD}_{0}$and ${SD}_{1}$represent the standard deviations of MASI at baseline and at the endpoint, respectively. The variance of Cohen's d is given by

$$V_{d}=(\frac{1}{n}+\frac{d^{2}}{2n})2(1-\rho)$$

where n is the number of pre-post pairs. The standard error of d is ${SE}_{d}=\sqrt{V_{d}}$.

And Hedges’ $g=d\cdot J$, and its standard error ${SE}_{g}={SE}_{d}\cdot J$, where the correction factor $J=1-\frac{3}{4(n-1)-1}$.

Then the resulting Hedges’ $g$ and its standard error can be used as inputs of *generic inverse variance* meta-analysis in Review Manager.

**Between Group: Meta-Analysis to Compare Reduction between Groups**

The standard deviation of the reduction per group is $\sqrt{{SD}_{0}^{2}+{SD}_{1}^{2}-2\rho\cdot{SD}_{0}\cdot{SD}_{1}}$, where $\mathrm{SD}_{0}$ and $\mathrm{SD}_{1}$, are the standard deviations of MASl at the baseline and the endpoint.
